# Hyperactivity of Basal Ganglia in Patients With Parkinson's Disease During Internally Guided Voluntary Movements

**DOI:** 10.3389/fneur.2019.00847

**Published:** 2019-08-07

**Authors:** Veronika Filyushkina, Valentin Popov, Rita Medvednik, Vadim Ushakov, Artem Batalov, Alexey Tomskiy, Igor Pronin, Alexey Sedov

**Affiliations:** ^1^Russian Academy of Sciences, Semenov Institute of Chemical Physics, Moscow, Russia; ^2^Burdenko National Scientific and Practical Center for Neurosurgery, Moscow, Russia; ^3^National Research Centre “Kurchatov Institute”, Moscow, Russia; ^4^Department of Physics of Living Systems, Moscow Institute of Physics and Technology, Moscow, Russia

**Keywords:** fMRI, externally triggered movement, internally guided movement, basal ganglia, Parkinson's disease

## Abstract

The contribution of different brain areas to internally guided (IG) and externally triggered (ET) movements has been a topic of debate. It has been hypothesized that IG movements are performed mainly through the basal ganglia-thalamocortical loop while ET movements are through the cerebello-thalamocortical pathway. We hypothesized that basal ganglia activity would be modified in patients with Parkinson's disease during IG movement as compared with normal subjects. We used functional MRI (fMRI) to investigate the differences between IG and ET motor tasks. Twenty healthy participants and 20 Parkinson's disease patients (OFF-state) were asked to perform hand movements in response to sound stimuli (ET) and in advance of the stimuli (IG). We showed that ET movements evoked activation of a few large clusters in the contralateral motor areas: the sensorimotor and premotor cortex, supplementary motor area (SMA), insula, putamen, motor thalamus and ipsilateral cerebellum. IG movements additionally evoked activation of a large number of small clusters distributed in different brain areas including the parietal and frontal lobes. Comparison between the activity of Parkinson's disease patients and healthy volunteers showed few important differences. We observed that along with the activity of the posterior areas, an activation of the anterior areas of putamen was observed during IG movements. We also found hyperactivity of the ventral thalamus for both movements. These results showed that IG movements in PD patients were made with the involvement of both sensorimotor and associative basal ganglia-thalamocortical loops.

## Introduction

The internal-external control hypothesis proposed that the cerebellum, parietal lobe, and lateral premotor cortex (PMC) would dominate during externally-triggered (ET) movements, whereas the basal ganglia (BG) and supplementary motor area (SMA) would show a predominant involvement in internally-guided (IG) movements (1). More recent studies of functional brain imaging in humans and single cell recordings in monkeys showed preferential involvement of the medially located SMA in self-initiated movement and the lateral premotor cortex in externally cued movement (2, 3). An event-related fMRI study showed activation within the basal ganglia, especially in putamen, during IG movements only (4). IG tasks were also characterized by significant interactions within the basal ganglia–thalamo–motor (BGTM) loop (5).

Clinical and experimental data has suggested that bradykinesia or slowness of movement initiation in Parkinson's disease may reflect an impaired connection between the supplementary motor area and putamen (6). Later neuroimaging studies have reported hypoactivation in the contralateral putamen and SMA (2, 7). In contrast, Yu et al. showed that putamen-SMA functional connectivity is enhanced in patients with PD (8). Neuroimaging studies have reported decreased percentage of activation in the regions within the BGTM during IG tasks and enhanced or preserved activation within the cerebello-cerebral (CC) loop during ET tasks in PD (9). Later disturbance in functional connectivity in the motor loop was found during IG but not ET movements in PD patients (7). On the other hand, electrophysiological studies in PD showed that BGTM circuit is involved in the preparation of both IG and ET movements but CC loop involved in the preparation of IG movement only (10).

Thus, the contribution of basal ganglia in various aspects of human movement remains unclear. Considering that in patients with Parkinson's disease the loss of dopamine is predominantly in the posterior putamen, associated with the control of habitual behavior (11), we hypothesized that basal ganglia activity pattern should be modified or displaced into associative areas during IG but not ET movement. The aim of the present study was to compare activation areas in the basal ganglia and thalamus during ET and IG motor tasks in normal and PD subjects.

## Method

Twenty right-handed patients with Parkinson's disease (nine males, 11 females) and 20 age- and gender-matched right-handed healthy volunteers (11 males, nine females) participated in the study (Supplementary Table 1). The disease severity according to the unified Parkinson's disease rating scale (UPDRS)-III, without levodopa administration, ranged from 21 to 71 points, mean disease duration was 13.8 + 4.5 years. All patients did not take medicine for 12 h before the study (OFF-state). Informed consent was obtained from all subjects prior to their participation in the study. The study was approved by the Ethics Committee of the Burdenko National Medical Research Center of Neurosurgery (01/2018). We used a block design paradigm with two distinct ET and IG movements to investigate the differences between these conditions. Each condition lasted 30 s with 30 s rest and was repeated seven times in a session. In ET mode, subjects were asked to perform a simple repetitive movement (clenching a fist) in response to external audio stimuli with a constant period 0.75 s. In IG mode, subjects were asked to perform the same goal-directed movements in advance of the stimulus with a constant period 1.5 s. In this case, the stimuli serve as reward, and movements initiated by internal command. We chose a longer interval between stimuli to avoid automaticity of movements.

Imaging was performed on a 3-Tesla MR-scanner with an eight-channel head coil. The protocol included: (1) A T1-weighted sagittal 3D rapid gradient echo sequence for anatomical data (voxel size 1 × 1 × 1 mm), and (2) a T2 EPI echo planar sequence for functional images (voxel size 1.8 × 1.8 × 4 mm). fMRI and anatomical data analysis was performed with SPM software (http://www.fil.ion.ucl.ac.uk/spm/software/spm12/). First level analysis was performed using general linear models with contrasts: ET>Rest and IG>Rest (12). Second level analysis was calculated using a one-sample *t*-test (*p* < 0.05) corrected with FWE. For the comparison of activation between the tasks, a paired *t*-test model (*p* < 0.001) corrected with FDR was used. The fMRI analysis was performed at the whole-brain level. The basal ganglia and thalamus areas were analyzed with the mask using WFU PickAtlas SPM package (https://www.nitrc.org/projects/wfu_pickatlas/).

## Results

We observed that ET movements evoked activation in several brain areas: the contralateral pre-central and post-central gyri including the primary motor cortex (M1), the somatosensory cortex (PSC) and PMC (Table 1, Supplementary Figure 1). Cortical activation also affected the contralateral rolandic operculum (RO), insula, and SMA. Subcortical structures were represented mainly by the posterior putamen and ventral thalamus. In addition, we observed activation in both sides of the cerebellum. IG movements evoked activation within widely distributed networks in both hemispheres (Table 1, Supplementary Figure 1). Along with motor cortical areas, we observed activations in the ipsilateral inferior parietal lobule, supramarginal gyrus, superior frontal gyrus, insula, and frontal operculum. It is worth highlighting that there was activation of both sides of the SMA. Significant activations of subcortical structures were found in the ventral thalamic nuclei, pallidum, putamen, and anterior caudate. We also observed a few clusters of activation in the thalamus and putamen in the ipsilateral hemispheres as well as in the both sides of the cerebellum (Table 1, Supplementary Figure 1). IG>ET contrast indicated activation predominantly in the right hemisphere, with peak activation in the right insular area, SMA, superior frontal gyrus, frontal inferior operculum, and parietal inferior lobule as well as activation in the right cerebellar lobule VI (Supplementary Figure 3, Supplementary Table 2). Using opposite contrast we observed activation of several clusters in the contralateral pre-central and post-central gyri as well as in the cingulum and precuneus areas (Supplementary Figure 3).

**Table 1:** Localization of activated areas during ET and IG movements in control group and PD patients.

**Control subjects**							**PD patients**						
**Cluster**	**N voxels**	**Peak MNI coordinates**			**Lable (aal)**	**Mean T**	**Cluster**	**N voxels**	**Peak MNI coordinates**			**Lable (aal)**	**Mean T**
		**X**	**Y**	**Z**					**X**	**Y**	**Z**		
**ET MOVEMENT**													
1	863	40	22	58	Pre-central L	7,4	1	634	37	29	50	Post-central L	7,0
					Post-central L							Pre-central L	
					Parietal Inf L							Parietal Inf L	
					Frontal Sup L		2	176	61	42	10	Temporal Sup L	5,9
2	419	41	29	18	Temporal Sup L	6,8						SupraMarginal L	
					Rolandic Oper L							Rolandic Oper L	
					SupraMarginal L							Temporal Mid L	
					Post-central L							Post-central L	
					Heschl L		3	159	5	9	54	Supp Motor Area L	6,6
					Insula L							Cingulum Mid L	
3	171	7	7	58	Supp Motor Area L	6,3						Supp Motor Area R	
					Supp Motor Area R		4	90	67	26	6	Temporal Sup R	5,8
4	58	41	3	14	Insula L	6,2						Temporal Mid R	
					Rolandic Oper L		5	201	16	24	2	Thalamus L	6,0
5	393	14	22	2	Thalamus L	6,7						Putamen L	
					Putamen L							Hippocampus L	
					Insula L							Pallidum L	
					Pallidum L		6	188	10	59	22	Cerebellum 4 5 R	6,6
6	819	18	48	26	Cerebellum 4 5 R	7,6						Vermis 4 5	
					Cerebellum 6 R							Cerebellum 6 R	
					Vermis 4 5							Vermis 6	
					Vermis 6							Cerebellum 3 R	
					Fusiform R								
					Lingual R								
					Cerebellum 3 R								
					Vermis 7								
					Vermis 8								
					Cerebellum 4 5 L								
					Cerebellum 8 R								
					Vermis 3								
7	82	40,44	2,96	14	Cerebellum 6 L	6,2							
**IG MOVEMENT**													
1	485	7	7	58	Supp Motor Area L	6,3	1	559	35	31	50	Post-central L	6,9
					Supp Motor Area R							Pre-central L	
					Frontal Sup R							Parietal Inf L	
					Cingulum Mid R		2	394	5	9	54	Cingulum Mid L	6,8
					Cingulum Mid L							Supp Motor Area L	
2	320	54	10	6	Insula R	6,3						Supp Motor Area R	
					Frontal Inf Oper R							Cingulum Mid R	
					Rolandic Oper R							Cingulum Ant L	
					Putamen R							Frontal Sup Medial L	
3	218	40	22	58	Pre-central L	6,4	3	347	31	16	2	Insula R	6,0
					Post-central L							Frontal Inf Oper R	
					Frontal Sup L							Pre-central R	
4	175	46	41	46	Parietal Inf R	6,4						Rolandic Oper R	
					SupraMarginal R							Putamen R	
					Parietal Sup R							Frontal Inf Tri R	
5	55	37	1	66	Frontal Mid R	5,9	4	196	54	44	34	Parietal Inf R	6,0
					Frontal Sup R							SupraMarginal R	
					Pre-central R							Angular R	
6	44	40	41	38	Parietal Inf L	5,9	5	162	35	42	30	Frontal Mid R	6,1
7	26	54	10	2	Frontal Inf Oper L	6,0						Frontal Inf Tri R	
					Rolandic Oper L		6	96	48	1	6	Rolandic Oper L	6,8
					Temporal Pole Sup L							Temporal Sup L	
					Insula L							Insula L	
8	15	42	1	6	Insula L							Temporal Pole Sup L	
					Rolandic Oper L							Frontal Inf Oper L	
9	16	40	35	26	Frontal Inf Tri R	6,0	7	71	54	26	22	Temporal Sup L	5,7
					Frontal Mid R	5,8						SupraMarginal L	
10	188	14	22	2	Thalamus L	6,1						Rolandic Oper L	
					Putamen L		8	66	61	42	10	Temporal Sup L	5,9
					Caudate L							Temporal Mid L	
11	499	18	48	22	Cerebellum 4 5 R	6,7	9	50	44	48	6	Frontal Mid R	6,1
					Vermis 4 5							Frontal Mid Orb R	
					Cerebellum 6 R							Frontal Inf Orb R	
					Vermis 6		10	26	67	27	6	Temporal Sup R	6,0
					Vermis 8							Temporal Mid R	
					Fusiform R		11	334	14	22	2	Thalamus L	6,2
					Vermis 7							Putamen L	
					Cerebellum 3 R							Pallidum L	
12	219	31	61	26	Cerebellum Crus1 L	6,9	12	31	14	22	10	Thalamus R	5,7
							13	140	12	59	22	Vermis 4 5	6,3
												Vermis 4 5	
												Cerebellum 6 R	
												Vermis 6	
												Cerebellum 4 5 L	

ET movements evoked mostly the same clusters in PD patients (Table 1, Supplementary Figure 2). We observed activation in the contralateral pre-central and post-central gyri during ET movements in PD patients (Table 1, Supplementary Figure 2). It is worth highlighting that there was a smaller activity cluster volume in SMA. Activity clusters were also found in the centralis cingulate, RO, and supramarginal gyrus. Among the subcortical structures it is worth noting that there was activity of the contralateral ventral thalamus, pallidum, and posterior putamen. We also observed activity in the ipsilateral cerebellum. As in the control group, IG movements evoked a wider range of activation areas (Table 1, Supplementary Figure 2). We did not observe displacement of activity from the sensorimotor areas to SMA as seen in the control group. We also observed activation clusters in the angular gyrus and bilateral supramarginal gyrus. Subcortical structures were presented the in bilateral thalamus and contralateral putamen. Activity was also observed in both sides of the cerebellum. IG>ET contrast showed activity in the ipsilateral hemisphere in the parietal lobe, angular gyrus, supramarginal gyrus, inferior frontal gyrus, pre-central gyrus, insula, and SMA (Supplementary Figure 3, Supplementary Table 2). We also observed activation of the ipsilateral anterior putamen. Opposite contrast did not reveal any significant activation.

**Figure 1:**
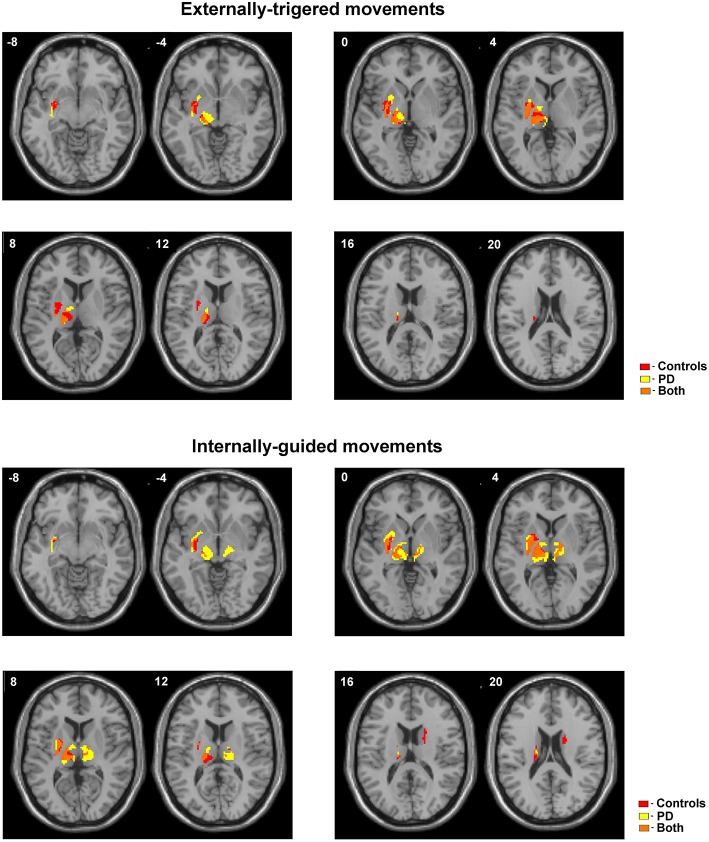
Activated areas during ET and IG movements superimposed on anatomic slice of averaged brain in control group and PD patients.

We observed a slight difference between basal ganglia and thalamic activities localization during ET movement and a robust localization difference during IG movements between PD and control. Figure 1 (bottom) shows additional activation in the anterior putamen, ventral thalamus, and subthalamic area in PD patients during ET movements. We also observed activity in the dorsal putamen in controls only. IG movements were characterized by more pronounced differences between PD and controls (Figure 1, top). In PD patients we observed hyperactivity in the contralateral putamen, ventral thalamus, and subthalamic areas. An activation cluster was also observed in the ipsilateral thalamus. At the same time, we observed activity in the ipsilateral caudate in controls only.

## Discussion

Internally generated and externally triggered movements are associated with different cortical activation patterns (2, 3, 13, 14). We showed distinctions between ET and IG motor behavior concerned the localization and cluster size of activated brain areas in healthy group. ET movements only activate the executive circuits of the motor system, i.e., the sensorimotor cortex, basal ganglia, thalamus, and cerebellum. These regions belong to the closed sensorimotor loop which is thought to support motor actions that require neither complex motor programming or the associative control system based on a feedback loop (15). In contrast, execution of IG movements is supported by widely distributed networks in both hemispheres, reflecting the fact that the internal command for movement initiation requires wider brain activation. We initially observed significant activation of the bilateral SMA which is thought to play a role in the initiation of movement (16). These results is in accordance with electrophysiological data showed that the amplitude of pre-movement event-related potentials (ERPs) over midline frontal structures as well as the amount of active SMA neurons are increased during internally driven with respect to externally triggered motor acts (2). Furthermore, along with the motor cortex, associative frontal and parietal areas were also engaged in IG movement performance.

Activation of the prefrontal cortex reflects the cognitive processes that underlie complex motor behaviors such as planning, preparation, and performing actions, as well as the processes involved in anticipating, predicting, and interpreting the consequences of actions. These findings suggest that IG motor actions are associated with not only sensorimotor activation but also activation of the associative loop involving higher-order integrative cortical areas which are strongly interconnected with the anterior striatum (15).

Despite the fact that basal ganglia connections with various parts of the cerebral cortex are well-studied, the contribution of these areas in various aspects of human movement remains unclear. Differences in participation of the basal ganglia and cerebellum loops are observed for planning and execution of IG and ET voluntary movements in healthy controls and PD patients (7, 10). Previous papers suggest disruption of cortico-striatal processing and preservation of relatively intact neural circuits that do not involve the basal ganglia in PD (5, 7). Electrophysiological study showed that deficit of self-initiated movement in PD patients is due to supplementary motor area underactivation (2).

We found activation in the main sensorimotor regions of the basal ganglia, namely the posterior putamen, pallidum, and ventrolateral thalamus, both during ET and IG movements in PD patients. At the same time, we found displacement of activation from the dorsolateral putamen in controls into the ventromedial direction in PD patients during ET movements. The most robust differences in basal ganglia were found during IG movements. We demonstrated hyperactivity in the putamen, including its anterior areas, and bilateral thalamus in PD patients. These results are contrary to previous data showed hypoactivation in the bilateral putamen in PD (7). According to the functional organization, anterior putamen with ventromedial prefrontal cortex are the part of the associative loop and play a significant role in goal-directed motor behavior (11, 17). On the other hand, the motor loop engages sensorimotor circuits and habitual performance, and includes the sensorimotor cortex and dorsolateral striatum, or posterior putamen. It has been theorized that imbalances of these two loops may lead to pathological conditions such as Parkinson's disease (11). We showed activation of both anterior and posterior putamen in PD patients unlike activation of only posterior putamen in the control group. We suppose that IG movements in PD could be controlled by an associative goal-directed network with the involvement of a wide range of cortical areas, which is activated in situations requiring non-routine decision making as in the self-initiated movements (2, 11). This could be a mechanism of compensation for disturbed sensorimotor control in PD patients. Further research of the SMA and basal ganglia network is needed for refinement of the pathological models of Parkinson's disease and improvement of treatment.

Our study has a limitation that should be mentioned. We used external reward stimuli in IG mode to unify paradigm. In the first few trials, some subjects could be wrong and performed movement with stimuli. We suppose that these trials do not significantly influence the results.

## Ethics Statement

The study was approved by the Ethics Committee of the Burdenko National Medical Research Center of Neurosurgery (01/2018).

## Author Contributions

AS, AT, and IP contributed conception and design of the study. VF, VP, AB, and VU organized the database. VF and RM performed the statistical analysis. AS wrote the first draft of the manuscript. VF, VP, AT, and VU wrote sections of the manuscript. All authors contributed to manuscript revision, read, and approved the submitted version.

### Conflict of Interest Statement

The authors declare that the research was conducted in the absence of any commercial or financial relationships that could be construed as a potential conflict of interest.

## Supplementary Material

Supplementary Figure 1 Statistical parametric maps of activated areas during externally triggered (ET) and internally guided (IG) movements in control groups. On the right—activated areas superimposed on anatomic slice of averaged brain.

Supplementary Figure 2 Statistical parametric maps of activated areas during externally triggered (ET) and internally guided (IG) movements in PD patients. On the right—activated areas superimposed on anatomic slice of averaged brain.

Supplementary Figure 3 Localization of activation areas using ET>IG and IG>ET contrast in control subjects and PD patients.

Supplementary Table 1 Clinical characteristics of Parkinson‘s disease patients.

Supplementary Table 2 Localization of activation areas using ET>IG and IG>ET contrast in control subjects and PD patients.
