# Supplementary figures and images for: Hyperactivity of Basal Ganglia in Patients With Parkinson's Disease During Internally Guided Voluntary Movements
Source: Front Neurol. 2019 Aug 7;10:847. doi: 10.3389/fneur.2019.00847 (PMC6692433; doi:10.3389/fneur.2019.00847)

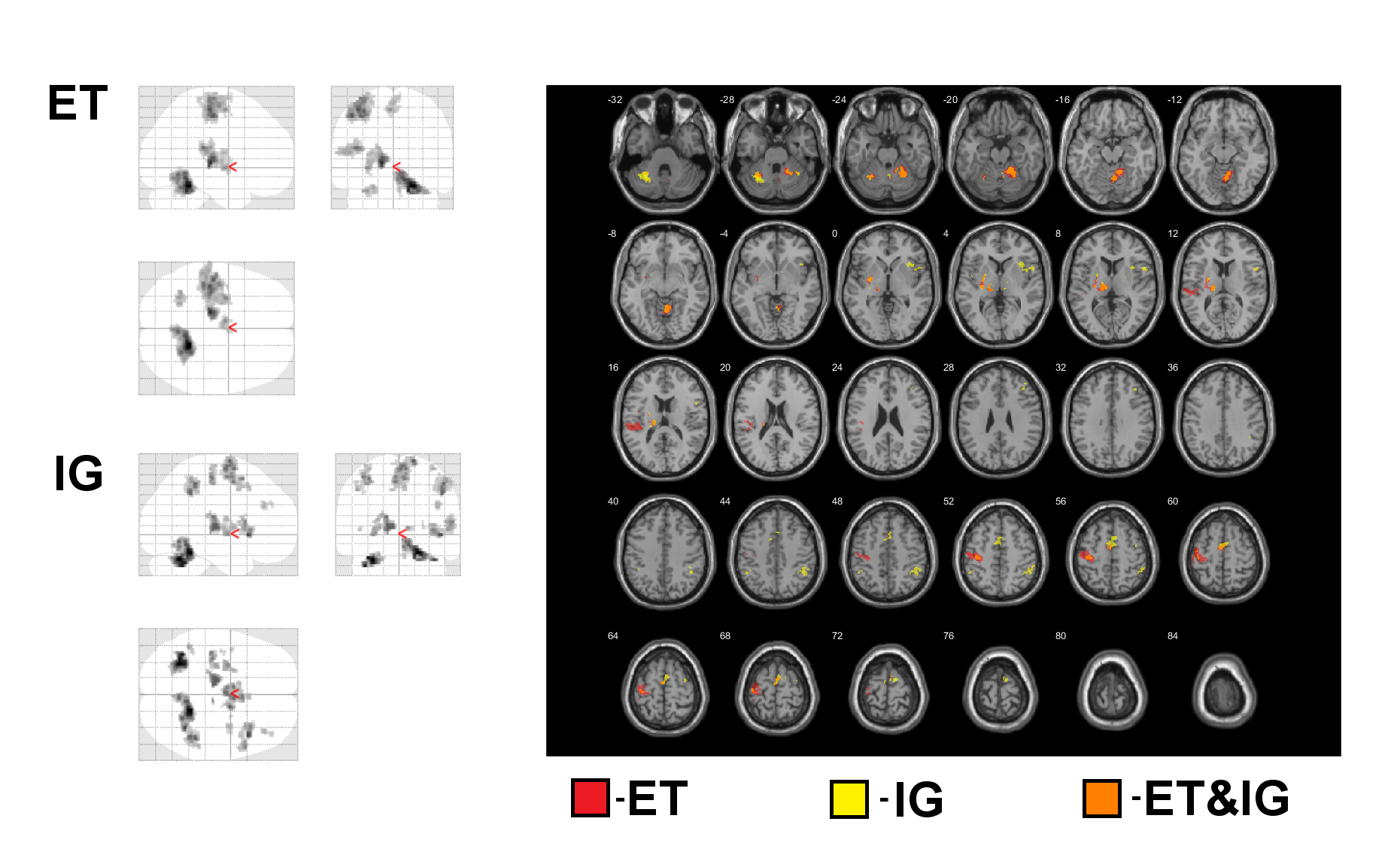

Supplement: Supplementary Figure 1 — Statistical parametric maps of activated areas during externally triggered (ET) and internally guided (IG) movements in control groups. On the right—activated areas superimposed on anatomic slice of averaged brain. [file Image_1.TIF]

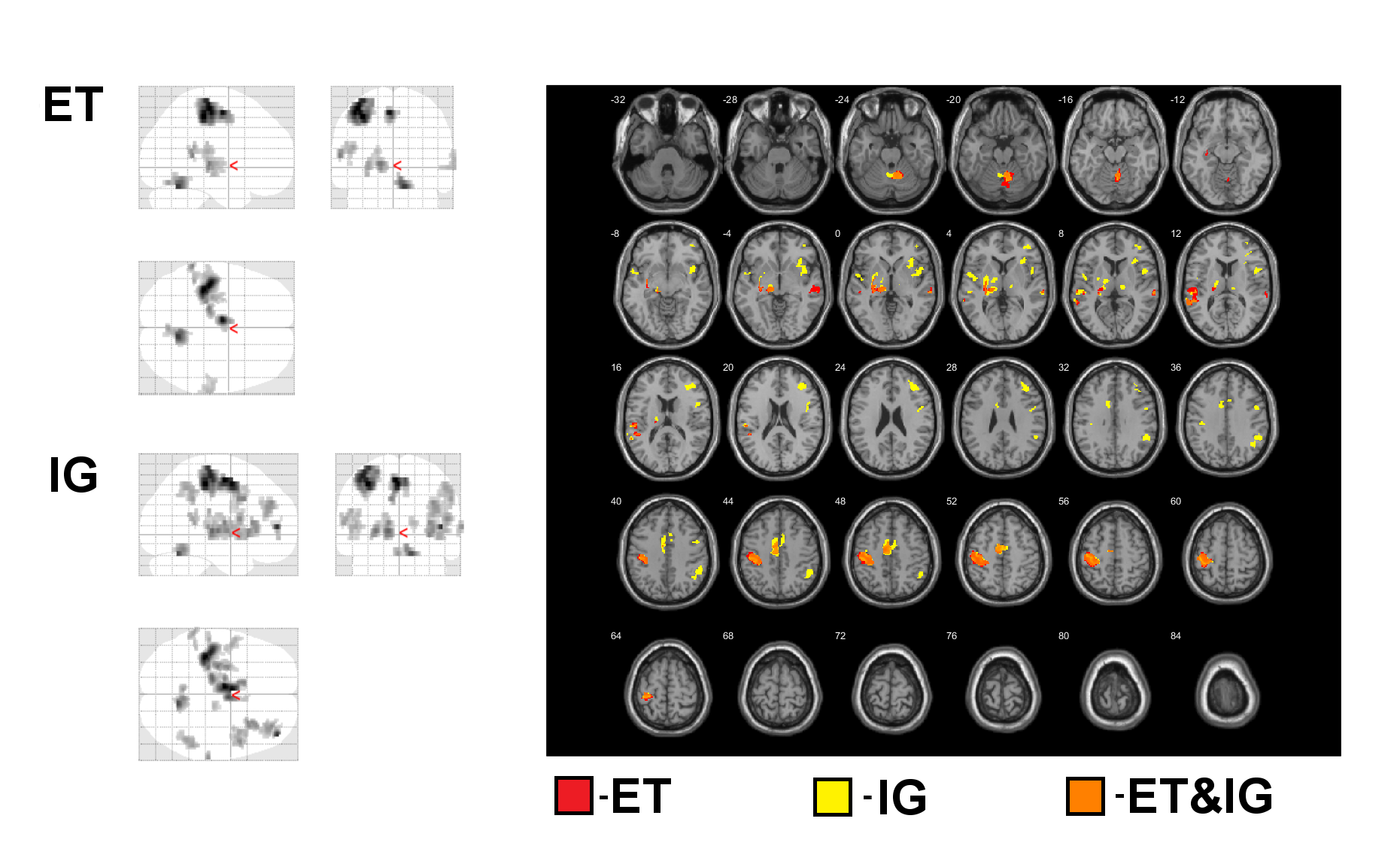

Supplement: Supplementary Figure 2 — Statistical parametric maps of activated areas during externally triggered (ET) and internally guided (IG) movements in PD patients. On the right—activated areas superimposed on anatomic slice of averaged brain. [file Image_2.TIF]

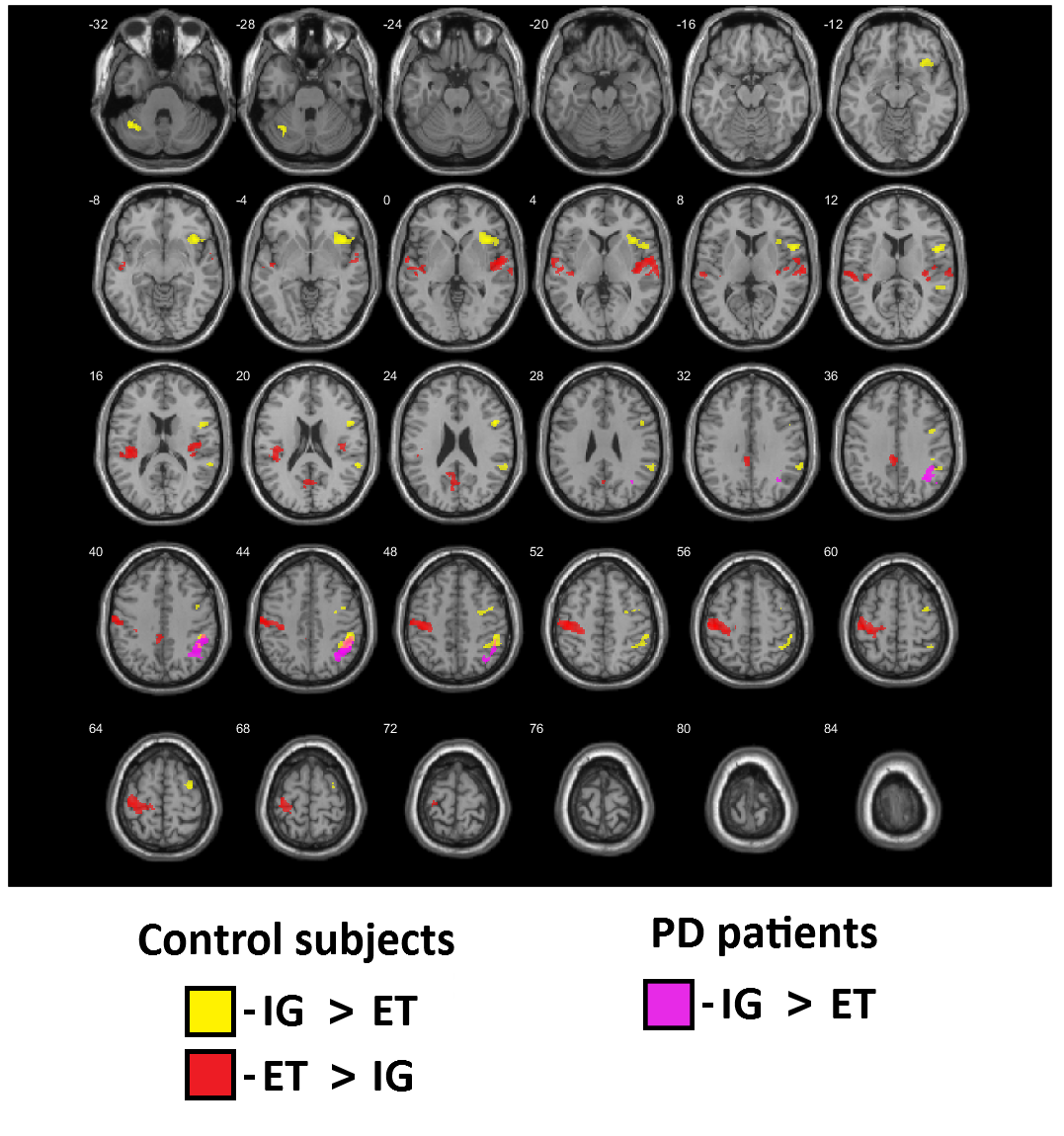

Supplement: Supplementary Figure 3 — Localization of activation areas using ET>IG and IG>ET contrast in control subjects and PD patients. [file Image_3.TIF]
