# Supplementary material for: Hyperactivity of Basal Ganglia in Patients With Parkinson's Disease During Internally Guided Voluntary Movements
Source: Front Neurol. 2019 Aug 7;10:847. doi: 10.3389/fneur.2019.00847 (PMC6692433; doi:10.3389/fneur.2019.00847)
Supplement: Supplementary Table 1 — Clinical characteristics of Parkinson‘s disease patients. [file Table_1.DOCX]

Supplementary Table. Localization of activation areas using ET>IG and IG>ET contrast in control subjects and PD patients.

| **ET > IG** | | | | | | | **IG > ET** | | | | | | |
| --- | --- | --- | --- | --- | --- | --- | --- | --- | --- | --- | --- | --- | --- |
| **Control subjects** | | | | | | | | | | | | | |
| **Cluster** | **N voxels** | **Peak MNI coordinates** | | | **Lable (aal)** | **Mean T** | **Cluster** | **N voxels** | **Peak MNI coordinates** | | | **Lable (aal)** | **Mean T** |
|  |  | X | Y | Z |  |  |  |  | X | Y | Z |  |  |
| 1 | 670 | -59 | -16 | 46 | Postcentral L | 4,4 | 1 | 416 | 40 | 20 | -6 | Insula R | 4,2 |
|  |  |  |  |  | Precentral L |  |  |  |  |  |  | Frontal Inf Oper R |  |
|  |  |  |  |  | Paracentral Lobule L |  |  |  |  |  |  | Rolandic Oper R |  |
|  |  |  |  |  | Parietal Inf L |  |  |  |  |  |  | Frontal Inf Tri R |  |
|  |  |  |  |  | SupraMarginal L |  |  |  |  |  |  | Frontal Inf Orb R |  |
| 2 | 419 | 54 | -9 | 2 | Temporal Sup R | 4,3 |  |  |  |  |  | Precentral R |  |
|  |  |  |  |  | Rolandic Oper R |  |  |  |  |  |  | Putamen R |  |
|  |  |  |  |  | Heschl R |  | 2 | 326 | 52 | -37 | 58 | Parietal Inf R | 4,0 |
|  |  |  |  |  | Insula R |  |  |  |  |  |  | SupraMarginal R |  |
|  |  |  |  |  | Postcentral R |  |  |  |  |  |  | Parietal Sup R |  |
|  |  |  |  |  | Temporal Pole Sup R |  |  |  |  |  |  | Temporal Sup R |  |
| 3 | 323 | -40 | -24 | 18 | Temporal Sup L | 4,1 |  |  |  |  |  | Postcentral R |  |
|  |  |  |  |  | Rolandic Oper L |  | 3 | 106 | 35 | -3 | 66 | Precentral_R | 4,0 |
|  |  |  |  |  | Insula L |  |  |  |  |  |  | Frontal_Sup_R |  |
|  |  |  |  |  | Heschl L |  |  |  |  |  |  | Frontal_Mid_R |  |
|  |  |  |  |  | SupraMarginal L |  | 4 | 87 | -39 | -56 | -30 | Cerebelum Crus1 L | 4,2 |
|  |  |  |  |  | Postcentral L |  |  |  |  |  |  | Cerebelum 6 L |  |
|  |  |  |  |  | Temporal Mid L |  |  |  |  |  |  |  |  |
| 4 | 84 | -1 | -61 | 22 | Precuneus L | 3,9 |  |  |  |  |  |  |  |
|  |  |  |  |  | Cuneus L |  |  |  |  |  |  |  |  |
|  |  |  |  |  | Precuneus R |  |  |  |  |  |  |  |  |
|  |  |  |  |  | Cingulum Post L |  |  |  |  |  |  |  |  |
|  |  |  |  |  | Calcarine L |  |  |  |  |  |  |  |  |
| 5 | 76 | -3 | -35 | 38 | Cingulum Mid L | 4,2 |  |  |  |  |  |  |  |
| **ET > IG** | | | | | | | **IG > ET** | | | | | | |
| **PD patients** | | | | | | | | | | | | | |
| **Cluster** | **N voxels** | **Peak MNI coordinates** | | | **Lable (aal)** | **Mean T** | **Cluster** | **N voxels** | **Peak MNI coordinates** | | | **Lable (aal)** | **Mean T** |
|  |  | X | Y | Z |  |  |  |  | X | Y | Z |  |  |
|  |  |  |  |  |  |  | 1 | 303 | 50 | -48 | 46 | Parietal Inf R | 4,3 |
|  |  |  |  |  |  |  |  |  |  |  |  | Angular R |  |
|  |  |  |  |  |  |  |  |  |  |  |  | SupraMarginal R |  |
|  |  |  |  |  |  |  |  |  |  |  |  | Parietal Sup R |  |
|  |  |  |  |  |  |  |  |  |  |  |  | Occipital Mid R |  |
