# Supplementary material for: Hyperactivity of Basal Ganglia in Patients With Parkinson's Disease During Internally Guided Voluntary Movements
Source: Front Neurol. 2019 Aug 7;10:847. doi: 10.3389/fneur.2019.00847 (PMC6692433; doi:10.3389/fneur.2019.00847)
Supplement: Supplementary Table 2 — Localization of activation areas using ET>IG and IG>ET contrast in control subjects and PD patients. [file Table_2.DOCX]

| Patient | Age (years) | Hoehn and Yahr | Disease duration  (years) | UPDRS III  OFF |
| --- | --- | --- | --- | --- |
| 1 (F) | 58 | 3 | 15 | 31 |
| 2 (F) | 63 | 3 | 15 | 47 |
| 3 (M) | 60 | 3 | 9 | 42 |
| 4 (M) | 43 | 2,5 | 8 | 21 |
| 5 (M) | 61 | 4 | 15 | 63 |
| 6 (M) | 41 | 3 | 17 | 23 |
| 7 (F) | 59 | 4 | 12 | 69 |
| 8 (F) | 67 | 4 | 9 | 49 |
| 9 (M) | 63 | 3 | 19 | 24 |
| 10 (F) | 63 | 3-4 | 17 | 65 |
| 11 (F) | 63 | 3 | 9 | 48 |
| 12 (M) | 45 | 2,5 | 9 | 32 |
| 13 (F) | 59 | 3 | 23 | 45 |
| 14 (F) | 62 | 3 | 18 | 41 |
| 15 (M) | 53 | 4 | 9 | 47 |
| 16 (M) | 65 | 3 | 14 | 23 |
| 17 (F) | 70 | 4 | 22 | 54 |
| 18 (M) | 50 | 3 | 13 | 29 |
| 19 (F) | 52 | 3 | 12 | 71 |
| 20 (F) | 55 | 3 | 10 | 44 |

Supp. Table 1. Clinical characteristics of Parkinson`s disease patients

F-Female; M-male; UPDRS III-unified Parkinson`s disease rating scale Part III: clinician-scored monitored motor evaluation; OFF-off levodopa medication.
